# The number of risk factors increases the recurrence events in ischemic stroke

**DOI:** 10.1186/s40001-022-00768-y

**Published:** 2022-08-02

**Authors:** Cep Juli, Henhen Heryaman, Arnengsih, Eng-Tat Ang, Irma Ruslina Defi, Uni Gamayani, Nur Atik

**Affiliations:** 1 Doctoral Program, Faculty of Medicine, Padjadjaran University, Bandung, Indonesia; 2 Department of Neurology Dr. Hasan Sadikin General Hospital/Faculty of Medicine, Padjadjaran University, Bandung, Indonesia; 3 Department of Anatomy, Yong Loo Lin School of Medicine, National University of Singapore, Bandung, Singapore; 4 Department of Physical Medicine and Rehabilitation, Faculty of Medicine, Dr. Hasan Sadikin General Hospital, Padjadjaran University, Bandung, Indonesia; 5 Department of Biomedical Sciences, Faculty of Medicine, Universitas Padjadjaran, Bandung, Indonesia

**Keywords:** Age, Number of risk factors, Recurrent ischemic stroke

## Abstract

**Purpose:**

Stroke is a significant cause of disability worldwide and is considered a disease caused by long-term exposure to lifestyle-related risk factors. These risk factors influence the first event of stroke and recurrent stroke events, which carry more significant risks for more severe disabilities. This study specifically compared the risk factors and neurological outcome of patients with recurrent ischemic stroke to those who had just experienced their first stroke among patients admitted to the Hospital.

**Patients and methods:**

We observed and analyzed 300 patients’ data who met the inclusion and exclusion criteria. This retrospective observational study was conducted on consecutive acute ischemic stroke patients admitted to the top referral hospital, West Java, Indonesia. The data displayed are epidemiological characteristics, NIHSS score at admission and discharge, and the type and number of risk factors. Data were then analyzed using appropriate statistical tests.

**Results:**

Most patients had more than one risk factor with hypertension as the most frequent (268 subjects or 89.3%). In patients who experienced ischemic stroke for the first time, the average National Institutes of Health Stroke Scale (NIHSS) score was lower (6.52 ± 3.55), and the alteration of NIHSS score was higher (1.22 ± 2.26) than those with recurrent stroke (6.96 ± 3.55) for NIHSS score and 1.21 ± 1.73 for alteration of NIHSS score). We processed the data with statistical analysis and showed a positive correlation between age (*P* < 0.05) and the number of risk factors (*P* < 0.001) in the recurrent ischemic stroke group.

**Conclusions:**

Age and the number of risk factors correlate with recurrent ischemic strokes.

## Introduction

Stroke has become the main cause of disabilities and mortality throughout the world [1]. Ischemic stroke contributes 85% of the total stroke cases, with thrombotic ischemic stroke as the primary pathology, in which the main underlying process is atherosclerosis [2–4]. Stroke is a damaging health problem that leads to an enormous treatment cost, creating additional difficulties considering that a higher number of stroke patients is thought to survive in developing countries [5, 6].

Since stroke is considered a disease caused by long-term exposure to lifestyle-related risk factors, modification of these risk factors will highly influence the event of stroke and stroke-related disability level [7]. It has been acknowledged that the risk factors for ischemic thrombotic stroke consist of modifiable and nonmodifiable risk factors, with gender, age, ethnicity, and race as the nonmodifiable factors and hypertension, dyslipidemia, diabetes mellitus, and smoking as the modifiable risk factors [7, 8].

Risk factors and lifestyles vary from one country to another, making explorations of the stroke risk factors, along with their clinical features, an essential measure to determine the pattern of stroke in specific populations. This is especially paramount in patients with recurrent stroke events. Recurrent strokes are dangerous threats and can be fatal; thus, we should avoid them. Prevention of recurrent strokes is a valuable process, and data need to be available to support efforts to minimize the incident that can avert high treatment and rehabilitation costs [9].

Stroke severity can be clinically assessed based on neurological disorder parameters [7]. Because of the long-term disability effect associated with stroke, making an accurate early prediction of future functional ability is imperative. This will then determine the therapeutic objectives that are adjusted to the needs of the patient as well as provide the prognostic overview of the patient's future condition [10].

The NIHSS is commonly used in the clinical practice of acute stroke to evaluate the neurological outcome. [10, 11]. It is often used as a standardized instrument to evaluate the severity of neurological deficits in patients admitted to the emergency room and stroke unit. NIHSS has predictive validity, because its initial score is a strong predictor for stroke complications during hospitalization and neurological outputs in the 3-month post-stroke. It is responsive to changes and can measure disorders across the stroke severity range [11]. A lower NIHSS score during the initial phase of acute ischemic stroke independently correlates with good neurological outcomes [10].

In this study, we want to compare risk factors and neurologic outcomes in patients with a first ischemic stroke with those with recurrent ischemic stroke.

## Materials and methods

### Subject selection and study design

This study was a cross-sectional analytical observational using retrospective data from 300 medical records admitted to the Neurological Department of Dr. Hasan Sadikin General Hospital, the top referral hospital in West Java, Indonesia, from November 2018 to April 2020. The type of stroke was classified according to the TOAST classification, whereas the subtypes within the inclusion criteria were extensive artery atherosclerosis and small artery occlusion. Stroke severity was stratified based on NIHSS score as follows: 1–4 mild, 5–15 moderate, and more than 15 was a severe stroke [10, 11]. All patients did not treat with thrombolysis or thrombectomy [3]. The inclusion criteria included acute ischemic stroke patients with infarction that matched the thrombotic features (atherothrombotic, thromboembolic, and lacunar) on a head CT scan without contrast. Meanwhile, the exclusion criteria were the presence of etiology of cardioembolic strokes, such as atrial fibrillation and myocardial infarction, and also features of cardioembolic infarction on CT scan [12–14]. Data was then analyzed to know the relationship of a risk factor with recurrent ischemic stroke events and compare their neurological outcomes using an appropriate statistical test.

### Statistical analysis

Categorical data are presented in number, frequency, and percentage, while numerical data are presented in mean, standard deviation, and range. Data analysis was started with the numerical data normality testing using the Kolmogorov–Smirnov test. The unpaired *T* test was used to compare the means between numerical data with normal distribution, while the Mann–Whitney test was used for data that were not normally distributed. For categorical data, the Chi-square test, with Kolmogorov–Smirnov dan exact Fisher tests, were used as alternatives if the requirements for the Chi-square test were not met. Significance was assessed using the *P* value with *p* ≤ 0.05 considered statistically significant.

## Results

During the study period, data from 300 patients with ischemic stroke were obtained. Slightly more males (51.7%) were included in the study, with most subjects were  < 60 years (64%). The average NIHSS score on admission was 6.71±3.540, while the score at discharge was 5.50 ± 3.135. The majority of patients were admitted (62.0%) and discharged (53%) with moderate NIHSS scores (Table 1). Hypertension was identified as the most frequent risk factor among these patients (89.3%), and most patients had three risk factors (35.3%) (Table 2). During the acute phase of ischemic stroke, patients were given antiplatelet aggregation, antihypertensive and diabetes therapy, cholesterol, and uric acid lowering drugs, depending on the risk factors. Patients are also advised to quit smoking. Management is following the guidelines widely used in the field of neurology [15, 16].

**Table 1:** Ischemic stroke patient characteristics

Variable	*N* = 300
Gender	
Male	155 (51.7%)
Female	145 (48.3%)
Age	
<60 years	192 (64.0%)
≥60 years	108 (36.0%)
NIHSS score at admission	
Mean	6.71 ± 3.540
Median	6.00
Range (min–max)	1.00–21.00
NIHSS degree at admission	
Minor	101 (33.7%)
Moderate	186 (62.0%)
Severe	13 (4.3%)
NIHSS score at discharge	
Mean	5.50 ± 3.135
Median	5.00
Range (min–max)	0.00–16.00
NIHSS degree at discharge	
Minor	139 (46.3%)
Moderate	159 (53.0%)
Severe	2 (0.7%)

**Table 2:** Risk factors in ischemic stroke patients

Variable	*N* = 300
Hypertension	
Yes	268 (89.3%)
No	32 (10.7%)
DM	
Yes	75 (25.0%)
No	225 (75.0%)
Dyslipidemia	
Yes	173 (57.7%)
No	127 (42.3%)
Smoking	
Yes	58 (19.3%)
No	242 (80.7%)
Hyperuricemia	
Yes	30 (10.0%)
No	270 (90.0%)
Age	
≥60 years	108 (36.0%)
<60 years	192 (64.0%)
Number of risk factor	
1	32 (10.7%)
2	88 (29.3%)
3	106 (35.3%)
4	57 (19.0%)
≥5	17 (5.7%)

The relationship between the number of risk factors and age with the recurrence of ischemic stroke was considered to be statistically significant (*P* < 0.05) (Table 3).

**Table 3:** Comparison of risk factors between recurrent stroke patients and first-ever stroke patients

Variable	Group		*P* value
	Recurrent stroke	First-ever stroke	
	*N* = 128	*N* = 172	
Hipertension			0.078
Yes	119 (93.0%)	149 (86.6%)	
No	9 (7.0%)	23 (13.4%)	
DM			0.590
Yes	30 (23.4%)	45 (26.2%)	
No	98 (76.6%)	127 (73.8%)	
Dyslipidemia			0.965
Yes	74 (57.8%)	99 (57.6%)	
No	54 (42.2%)	73 (42.4%)	
Smoking			0.711
Yes	26 (20.3%)	32 (18.6%)	
No	102 (79.7%)	140 (81.4%)	
Hyperuricemia			0.062
Yes	8 (6.3%)	22 (12.8%)	
No	120 (93.8%)	150 (87.2%)	
Age			
≥60 years	55 (43.0%)	53 (30.8%)	0.030^a^
<60 years	73 (57.0%)	119 (69.2%)	
Number of risk factors			0.0001^b^
Median	3.00	2.00	
Range (min–max)	2.00–6.00	1.00–4.00	
Risk factors			0.0001^b^
1	0 (0.0%)	32 (18.6%)	
2	19 (14.8%)	69 (40.1%)	
3	52 (40.6%)	54 (31.4%)	
4	40 (31.3%)	17 (9.9%)	
≥5	17 (13.3%)	0 (0.0%)	

^a^Chi-square test

^b^Mann–Whitney test

Significance at *P* < 0.05

We showed that the most infarction located in basal ganglia. The statistical analysis of the relationship between infarction location and recurrent ischemic stroke and first-ever ischemic stroke showed that P was more than 0.05 (*P* > 0.05), reflecting a statistically insignificant difference (Table 4).

**Table 4:** Comparison of infarction location between recurrent stroke patients and first-ever stroke patients

Variable	Group		*P* value
	Recurrent stroke	First-ever stroke	
	*N* = 128	*N* = 172	
Thalamus			0.703
Yes	2 (1.6%)	5 (2.9%)	
No	126 (98.4%)	167 (97.1%)	
Lateral periventricular			0.220
Yes	37 (28.9%)	39 (22.7%)	
No	91 (71.1%)	133 (77.3%)	
Pons			0.721
Yes	9 (7.0%)	14 (8.1%)	
No	119 (93.0%)	158 (91.9%)	
Basal ganglia			0.525
Yes	68 (53.1%)	85 (49.4%)	
No	60 (46.9%)	87 (50.6%)	
Temporoparietal lobe			0.398
Yes	1 (0.8%)	4 (2.3%)	
No	127 (99.2%)	168 (97.7%)	
Parietal lobe			0.795
Yes	6 (4.7%)	7 (4.1%)	
No	122 (95.3%)	165 (95.9%)	
Occipital lobe			0.427
Yes	1 (0.8%)	0 (0.0%)	
No	127 (99.2%)	172 (100.0%)	
Temporal lobe			0.474
Yes	2 (1.6%)	6 (3.5%)	
No	126 (98.4%)	166 (96.5%)	
Parietoocipitali lobe			0.181
Yes	2 (1.6%)	0 (0.0%)	
No	126 (98.4%)	172 (100.0%)	
Frontal lobe			0.474
Yes	2 (1.6%)	6 (3.5%)	
No	126 (98.4%)	166 (96.5%)	
Frontotemporal lobe			1.000
Yes	1 (0.8%)	2 (1.2%)	
No	127 (99.2%)	170 (98.8%)	
Internal capsule			0.845
Yes	12 (9.4%)	15 (8.7%)	
No	116 (90.6%)	157 (91.3%)	

The relationship between the average NIHSS score and NIHSS degree at admission and discharge in recurrent ischemic stroke and first-ever ischemic stroke groups was statistically insignificant (*P* > 0.05) (Table 5).

**Table 5:** Comparison between NIHSS and the occurence of recurrent stroke and first-ever stroke

Variable	Group		*P* value
	Recurrent stroke	First-ever stroke	
	*N* = 128	*N* = 172	
NIHSS score at admission			0.264
Mean ± std	6.96 ± 3.55	6.52 ± 3.53	
Median	6.00	6.00	
Range (min–max)	2.00–18.00	1.00–21.00	
NIHSS degree at admission			0.367
Minor	38 (29.7%)	63 (36.6%)	
Moderate	83 (64.8%)	103 (59.9%)	
Severe	7 (5.5%)	6 (3.5%)	
NIHSS score at discharge			0.393
Mean ± std	5.75 ± 3.28	5.31 ± 3.02	
Median	5.00	5.00	
Range (min–max)	1.00–16.00	0.00–16.00	
NIHSS degree at discharge^a^			0.938
Minor	58 (45.3%)	81 (47.1%)	
Moderate	69 (53.9%)	90 (52.3%)	
Severe	1 (0.8%)	1 (0.6%)	

The comparison of NIHSS alteration between recurrent ischemic stroke and first-ever stroke patients resulted in *P* > 0.05. This means that no statistically significant association was found between variables (Table 6).

**Table 6:** Comparison of NIHSS alteration between recurrent stroke and first-ever stroke patients

Variable	Group		*P* value
	Recurrent stroke	First-ever stroke	
	*N* = 128	*N* = 172	
Alteration of NIHSS			0.663
Mean ± std	1.21 ± 1.73	1.22 ± 2.26	
Median	0.00	0.00	
Range (min–max)	8.00–1.00	12.00–4.00	

## Discussion

Along with the increase in life expectancy, ischemic stroke with high morbidity has become an important public health problem. Although various studies are available, including studies on clinical characteristics and topography of ischemic stroke with different results, only a few studies have evaluated the risk factors and their relationship with neurological outcome in patients by comparing patients with recurrent ischemic stroke to patients with first-ever ischemic stroke [17–20].

In our study, age is an essential nonmodifiable risk factor for all patients with ischemic stroke and significant independent predictors of recurrent strokes. Stroke is an ageing-related disease with an incidence that increases dramatically with age [17]. The incidence increases twice for every decade after 55 [18, 19]. About three-quarters of all stroke events occur in people aged  ≥ 65 years. With ageing, brain microcirculation and macrocirculation undergo structural and functional changes. The microcirculation changes associated with increasing age are allegedly mediated by endothelial dysfunction and impaired brain autoregulation, and neurovascular coupling. Endothelial dysfunction increases nerve inflammation, disturbance of cerebral autoregulation leading to microvascular injuries, and impaired neurovascular coupling that decreases the cortical function [20].

More male patients were included in our study, both in the recurrent ischemic stroke group and the first-ever ischemic stroke group. Estrogen is protective in many tissues, including the brain, adipose tissue, heart, and blood vessels. In the context of ischemic stroke, estrogen has a highly neuroprotective nature in various levels, including suppressing the risk factor for stroke pathology through the anti-atherogenic effect in blood vessels and adipogenesis regulation. Estrogen also improves the stroke pathology through vasodilation of the coronary arteries and directing the nerve protection of the brain and glial cells during ischemia [21].

Gender difference in the epidemiology of ischemic stroke is age-dependent because of its influence on stroke risk and neurological outcome changes with age. In childhood and early adulthood, men have a higher incidence of ischemic stroke and worse functional outcomes than women. At middle age, the rate of ischemic stroke begins to rise among women, with the onset of menopause and the diminishing female sex hormones. After median age, the rate of stroke increases in women, with some reports showing higher stroke events in older women (age > 85 years) than older men [21].

In recurrent and first-ever ischemic strokes, hypertension is the most prominent modifiable risk factor, followed by dyslipidemia, diabetes, smoking, and hyperuricemia. The results of this study are the same as our previous research [22]. The mechanisms can explain the harmful effects of hypertension, dyslipidemia, DM, hyperuricemia, and smoking on the incidence of ischemic stroke. Hypertension influences cerebrovascular autoregulation by changing the mechanical characteristics of the brain blood vessels through remodeling and hardening and affecting the myogenic tone. Its changes in autoregulation specifically damage the periventricular white matter located in the border area between different arterial regions [4, 9, 23].

Dyslipidemia plays a complex role in cerebrovascular diseases [16]. There is a solid and direct relationship between total cholesterol, LDL, and ischemic stroke. Oxidized cholesterol, especially LDL, triggers inflammation and forms plaque on the blood vessel wall, blocking the arterial blood flow [24]. DM is associated with increased coagulation factors and hyperinsulinemia, which are essential in developing microangiopathy strokes. Macroangiopathy infarction occurs, because DM accelerates the atherosclerotic process of the large cerebral arteries[9]. Elevated uric acid levels are associated with the incidence and development of atherosclerosis. Uric acid can activate inflammasome proteins causing cell damage [25]. Most smokers experience stroke since smoking affects thrombosis and facilitates platelet aggregation by causing an imbalance between cerebral blood vessel coagulation and abnormal fibrinolysis. It changes the function of the blood–brain barrier and interferes with normal endothelial cell function [26].

We found that the variable differentiating the recurrent ischemic stroke from the first-ever ischemic stroke is the number of risk factors present. Recurrent ischemic stroke patients have more risk factors than patients who experience stroke for the first time. This is following previous research. A global population-based study has identified ten risk factors contributing to 88% of stroke risks. Since most individuals have several risk factors, they significantly increase the incidence of stroke [20, 27]. One explanation is that patients with multiple risk factors are difficult to control their risk factors; thus, they would be susceptible to recurring strokes. Stroke is stated to be a result of long-term exposure to co-existing risk factors [28]. Another factor is related to the pathological process underlying ischemic stroke caused by atherosclerosis. Thrombus formation in ruptured atherosclerotic plaques is the pathological basis of acute arterial thrombotic events [29, 30]. Several risk factors play an essential role in developing atherosclerosis and plaque instability [31–33].

No significant difference is found in the location of infarction between recurrent ischemic stroke patients and first-ever ischemic stroke patients. Infarction of thrombotic ischemic stroke is most numerous in the subcortical area, mainly in the basal ganglia [12]. In contrast to cardioembolic infarcts, which often cause large-sized infarcts in the cortical area, including malignant infarcts in the middle cerebral artery [14, 34, 35]. Lipohyalinosis and medial hypertrophy secondary to atherosclerosis mainly occur in the lenticulostriate arteries of the media cerebral arteries and the perforating arteries of the anterior cerebral arteries (known as recurrent Heubner arteries). These two arteries transport blood to the basal ganglia [12].

Over the past decade, radiological findings have been extensively studied to predict recurrent strokes. However, most imaging studies investigate different study populations and outcome definitions, making it difficult to draw conclusions that can be generalized. When considering imaging features as predictors, moderate evidence was found for the relationship between recurrent ischemic stroke and old ischemic lesions. Although white matter lesions are associated with stroke recurrence, the role of imaging predictors is less established and has only been investigated for MRI. Further research is needed on imaging modalities to visualize the characteristics and causes of recurrent ischemic stroke [34].

A High NIHSS value during acute ischemic stroke is one factor that plays a role in the incidence of recurrent stroke [36]. NIHSS values are associated with thrombogenic plaque levels and a reflection of reperfusion, which may result in a higher risk of recurrence of acute ischemic stroke [37, 38]. In our study, although the mean and alteration of NIHSS values in first-ever stroke patients were better than the values in recurrent ischemic stroke patients , the difference is not statistically significant. Many factors affect the neurological outcome. In the acute phase of stroke, the strongest predictors of neurological outcome are stroke severity and age. Stroke severity can be evaluated by looking at nerve damages and observing the size and location of infarction on MRI or head CT. Other important factors that affect neurological outputs are epidemiological factors, comorbid conditions, stroke complications, and ischemic stroke mechanisms [7].

Recovery from an acute ischemic stroke is a multifaceted process, ranging from hours to months. In the acute phase of stroke, it is difficult to predict the final recovery rate due to the magnitude of heterogeneity during recovery in the first 3 months after a stroke. The longer the physiological assessment time from the stroke event, the better and more specific the prediction. For example, neurological conditions on day 4 predict better long-term neurological outcomes than those on the first day of ischemic stroke [39].

Our study has limitations, because it is a single-site, hospital-based study. Therefore, a more extensive multicenter study is needed. Based on the results of this study, we recommend further research in screening for risk factors to prevent or minimize the risk of stroke recurrence. The prospective ongoing studies are also necessary to assess the neurological outcome with existing modalities.

## Conclusions

Our study showed that age and the number of risk factors correlate with recurrent ischemic strokes.

## Data Availability

The authors confirm that the data supporting the findings of this study are available within the article.
